# Supplementary material for: Large-scale computational modelling of the M1 and M2 synovial macrophages in rheumatoid arthritis
Source: NPJ Syst Biol Appl. 2024 Jan 26;10:10. doi: 10.1038/s41540-024-00337-5 (PMC10811231; doi:10.1038/s41540-024-00337-5)
Supplement: Supplementary file 1 — Supplementary material [file 41540_2024_337_MOESM1_ESM.pdf]

Large-scale computational modelling of the M1 and M2 synovial macrophages in Rheumatoid Arthritis.

Supplementary Data

Supplementary Table 1. The list of differentially expressed genes present in the RA M1 macrophage model that we identified using literature search and omics data analysis. The first column contains the DEGs HGNC names. The second and fifth columns contain their corresponding Boolean values observed in GSE97779 dataset and literature respectively.

DEG	Boolean value in GSE97779	Adjusted p_value	logFC	Boolean value based on literature	Reference
C5A				1	29220376
PRKCD	1	0,0008	0,86		
BAD	0	0,02492	-0,42		
STAT2				1	27626941
IRF9	1	0,00987	0,65	1	27626941
INHBA				1	13130463
SMAD4	0	0,012	-0,46		
CASP1	1	0,03031	0,71		
SIRT1	0	0,0284	-0,61	0	25799392
CCL21				1	21225692
PRKCQ	1	0,0037	1,53		
INHBB				1	26359667
TRAF6	0	0,00612	-0,73		
SMAD7	1	7,8882E-05	2,17		
INPP5A	1	0,0091	0,9		
DUSP1	1	5,59E-07	4,17		
PRKG1	1	0,00311	2,17		
ACVR2A	0	0,01746	-0,79		
ACVR2B	0	0,00045	-2,85		
BCL2	1	0,01417	1,67		
BCL2L1				1	28118944
BCL2L11	1	0,0833	1,78		
BCL3	1	0,04434	0,42		
BIRC2	1	0,00425	0,594		
C5AR1				1	29220376
CASP3	0	0,00542	-0,86		
CASP7	1	0,00655	0,96		
CCL2				1	33330982
CCR2	1	7,69E-07	5,85		

CFLAR	1	0,0029	0,8	1	12228167
CSF2RA	1	0,00287	1,11	1	24936585
CSF2RB	1	0,00099	0,93	1	24936585
CXCL1				1	7561066
FAS	1	0,0005	2,45		
FOS	1	2,99E-08	3,93	1	27626941
GNA13	1	0,00017	1,62		
GNAI3	1	0,00098	0,72		
GNB1	1	0,00507	0,47		
HLA-B	1	0,02221	0,42		
HRAS	1	0,00388	0,94		
IFNA1	1	0,00831	2,43		
IFNB				1	15878901
IFNGR1	1	0,01789	1,35	1	25708927
IFNGR2	1	0,00415	0,67	1	25708927
IL11				1	29327326
IL12RB1	1	0,00343	1,29		
IL18				1	10562301
IL18R1	1	0,00057	3,64		
IL1RAP	1	0,00018	2,73		
IL23				1	25799392
IL6	1	0,02477	1,56		
IL6ST	1	0,01567	0,56		
IL8				1	10491366
IRF7	1	1,89337E-05	2,44	1	22614743
JAK1	1	0,00433	1		
JAK2	1	0,0011	2,3		
LILRB1	1	0,0015	0,96		
MAP2K1	1	0,0001	1,12		
MAP2K2	1	0,00845	0,64		
MAP2K3	1	0,00258	1,03		
MAP2K6	1	0,02686	1,78		
MAPK14	0	0,0243	-0,61		
MAPK3				1	17907188
MAPK8	1	0,00374	0,71		
MAPKAPK2	1	0,00399	0,78		
MCL1	1	0,00118	1,39	1	17009247
MDM2	0	0,01706	-1,06		
MYC	1	0,00027	1,66		
NFAT5	1	0,01494	0,97		
NFKB1	1	0,00235	0,89	1	8630106
NFKBIA	1	0,00021	0,98		
NFKBIE	1	0,01037	0,79		
NLRP3	1	8,50E-06	4,44		
OPN3	0	0,00517	-0,87		
PPIA	1	0,01626	1,05		

PTK2	1	0,00073	1,87		
PTPN6	1	2,86974E-05	1		
RAC1	1	0,03366	0,44		
RAF1	1	0,01113	1		
RBPJ	1	3,82155E-05	2,5		
RELA	1	0,01152	0,66		
SMAD2	0	0,01636	-0,45		
SOS1	1	0,01592	1,32		
STAT1	1	0,00057	2,22	1	22614743
STAT3	1	0,00043	0,89		
STAT4				1	10779770
STAT5B	1	0,00037	2,34		
SYK	0	0,03668	-0,38		
TLR1	1	1,21E-06	1,65		
TLR2	1	8,13E-07	2,2	1	15146415
TLR4	0	0,01061	-0,67		
TLR5	0	0,00032	-1,25		
TLR8	1	0,04753	0,68		
TLR9				1	26759164
TNF	1	7,84837E-05	2,5	1	2109776
TNFRSF11	1	0,01287	1,79		
TNFRSF1A				1	9189061
TRADD	1	0,03339	0,47		
TRAF3	0	0,00022	-1,14		
TRAM1	0	0,01224	-0,4		
XIAP	1	0,00215	1,12	1	19171073
PPP4C	1	0,00131	0,68		
biglycan_simple_molecule				1	19772831
DNA_simple_molecule				1	19772831
dsRNA_simple_molecule				1	19772831

Supplementary Table 2. List of nodes upstream the phenotypes of interest in the RA M1 macrophage model associated with their mean values over the fixpoints having the highest similarity score.

**Nodes**	**Mean values**
CSF2RA_CSF2RB_complex	1
RELA_NFKB1_NFKBIE_complex	1
TLR5	0
IRF9	1
STAT2	1

IKK1_phosphorylated	0,5
col4a4	0,66666667
PTPN6	1
PRKG1	1
ACVR2A_ACVR2B_complex	0
ACVR2A_ACVR2B_INHBA_complex	0
ACVR2A_ACVR2B_INHBB_complex	0
AP_1	1
AP_1_phosphorylated	1
apoptosis_M1_macrophage_phenotype	0
ASC	1
ASK1	1
BAD	0
BCL2_M1_macrophage__Mitochondria_membrane	1
BCL2_M1_macrophage__Mitochondria_membrane_active	0
Bcl2_rna	1
BCL2L1_M1_macrophage__Mitochondria	1
BCL2L1_M1_macrophage__Mitochondria_active	0
BCL3_rna	1
biglycan_simple_molecule	1
Bim	1
c_FOS_M1_macrophage___Cytoplasm	1
c_FOS_M1_macrophage___Cytoplasm_active	1
c_FOS_M1_macrophage___nucleus	1
c_JUN	1
c_JUN_phosphorylated_M1_macrophage___Cytoplasm	1
c_JUN_phosphorylated_M1_macrophage___nucleus	1
c_Myc_rna	1
c5a	1
C5a_C5aR1_complex	1
Casp1	1
CASP3	0
CASP7	0
CASP8	1
CCL2_CCR2_complex	1
CCL2_M1_macrophage___Extracellular_Space	1
CCL2_M1_macrophage___Secreted_components	1
CCl21	1
CCL21_CCR7_complex	1
CD40LG_ITGB1_ITGA1_complex	0,5
cFLIP	1
cIAP1	1
col4a5	0,66666667
CRKL_phosphorylated	1
CSF2_M1_macrophage___Extracellular_Space	1
CSF2_M1_macrophage___Secreted_components	1

CSF2RA_CSF2RB_CSF2_complex	1
CXCL1_CXCR1_complex	1
CXCL1_M1_macrophage___Extracellular_Space	1
CXCL1_M1_macrophage___Secreted_components	1
CXCR1	1
CXCR1_IL8_complex	1
CypA	1
DAXX	1
DNA_simple_molecule	1
dsRNA_simple_molecule	1
DUSP1	1
ECSIT	0,5
ERK1_phosphorylated_M1_macrophage___Cytoplasm	1
ERK1_phosphorylated_M1_macrophage___nucleus	1
FADD	1
FAS	1
FASL_FAS_complex	1
FASL_M1_macrophage___Extracellular_Space	1
FASL_M1_macrophage___Secreted_components	1
FOXO_M1_macrophage___Cytoplasm	1
FOXO_M1_macrophage___nucleus	1
gal	1
GAL_GALR2_complex	1
gamma_secretase_complex_complex	1
GNA12_GNA13_complex	1
GNAI3	1
GNB_GNG_GNAI3_complex	1
HLA_B_LILRB1_complex	1
HRAS	1
IFNa_M1_macrophage___Extracellular_Space	1
IFNa_M1_macrophage___Secreted_components	1
IFNAR1_IFNAR2_complex	1
IFNAR1_IFNAR2_IFNa_complex	1
IFNAR1_IFNAR2_IFNb_complex	1
IFNb_M1_macrophage___Extracellular_Space	1
IFNb_M1_macrophage___Secreted_components	1
IFNE	0,5
IFNE_IFNAR1_IFNAR2_complex	0,5
IFNg_M1_macrophage___Extracellular_Space	1
IFNg_M1_macrophage___Secreted_components	1
IFNGR1_IFNGR2_complex	1
IFNGR1_IFNGR2_IFNg_complex	1
IKK_complex	1
IKK1_IKK2_complex	0,5
IKK2_phosphorylated	1
IKKE_TBK1_complex	0,5

IKKE_TBK1_TRAF3_complex	0
IL1_IL1R_complex	1
IL11_IL11Ra_IL6ST_complex	1
IL12_M1_macrophage___Extracellular_Space	1
IL12_M1_macrophage___Secreted_components	1
IL12RB_complex	1
IL12RB_IL12_complex	1
IL18_IL18R1_complex	1
IL18_M1_macrophage___Cytoplasm	1
IL18_M1_macrophage___Extracellular_Space	1
IL18_M1_macrophage___Secreted_components	1
IL1B_M1_macrophage___Cytoplasm	1
IL1B_M1_macrophage___Extracellular_Space	1
IL1B_M1_macrophage___Secreted_components	1
AKT1	0
IL1R_complex	1
IL23_M1_macrophage___Extracellular_Space	1
IL23_M1_macrophage___Secreted_components	1
IL23R_IL12RB1_complex	1
IL23R_IL12RB1_IL23_complex	1
IL6_IL6R_IL6ST_complex	1
IL6_M1_macrophage___Extracellular_Space	1
IL6_M1_macrophage___Secreted_components	1
IL8_M1_macrophage___Extracellular_Space	1
IL8_M1_macrophage___Secreted_components	1
INHBA	1
INHBB	1
INPP5A	1
IRAK1	1
IRAK1_IRAK4_complex	1
IRAK4_phosphorylated	1
IRF3_phosphorylated	1
IRF7	1
ITGB1_ITGA1_col4a_complex	1
ITGB1_ITGA1_complex	1
JAK1	1
JAK1_JAK2_complex	1
JAK1_TYK2_complex	1
JAK2	1
JAK2_TYK2_complex	1
JNK1_phosphorylated_M1_macrophage___Cytoplasm	1
JNK1_phosphorylated_M1_macrophage___nucleus	1
Mcl1_rna	1
MDM2_phosphorylated	0
MEK1_phosphorylated	1
MEK2_phosphorylated	1

MEKK1	0,5
MK2_phosphorylated	1
MKK3_phosphorylated	1
MKK4_phosphorylated	1
MKK6_phosphorylated	1
MKK7_phosphorylated	1
MYD88	1
MYD88_TIRAP_TOLLIP_complex	1
NCID	1
NFAT5	1
NFKB1_TPL2_complex	1
NFKBIA_RELA_NFKB1_complex	1
NICD	1
NICD_CSL_SKIP_MAML1_ep300_complex	1
NIK	1
NLRP3	1
NLRP3_INFLAMMASOME_complex	1
notch1_JAG1_complex	1
OPN	0
osteoclastogenesis_M1_macrophage_phenotype	1
p15_rna	0
p21_rna	1
p300_SP1_complex	0
p38_MAP_KINASE_phosphorylated_M1_macrophage___Cytoplasm	0
p38_MAP_KINASE_phosphorylated_M1_macrophage___nucleus	0
p53_phosphorylated	1
PI3K	0
PIK3AP1_phosphorylated	0
PP4	1
Prkcd	1
PRKCQ	1
proliferation_survival_M1_macrophage_phenotype	1
PTK2	1
Rac1	1
RAF1	1
RELA_NFKB1_complex_M1_macrophage___Cytoplasm	1
RELA_NFKB1_complex_M1_macrophage___nucleus	1
RHOA	0
SHP2_GRB2_complex	1
Sirt1	0
SMAD2_phosphorylated	0
SMAD2_SARA_complex	0
SMAD2_SMAD4_complex	0
SMAD4	0
SMAD7	1

SOS1	1
Src	1
STAT1	1
STAT1_STAT1_complex	1
STAT1_STAT2_IRF9_complex	1
STAT3	1
STAT3_STAT3_complex	1
STAT4	1
STAT4_STAT4_complex	1
STAT5_CRKL_complex	1
STAT5_phosphorylated	1
SYK	0
TAB1	1
TAB2_phosphorylated	1
TAK1	1
TLR1_TLR2_biglycan_complex	1
TLR1_TLR2_complex	1
TLR2_TLR6_biglycan_complex	1
TLR2_TLR6_complex	1
TLR3_dsRNA_complex	1
TLR4_Md2_CD14_fibrinogen_complex	0
TLR7_TLR8_ssRNA_complex	1
TLR9_DNA_complex	1
TNF_M1_macrophage___Extracellular_Space	1
TNF_M1_macrophage___Secreted_components	1
TNF_TNFRSF1A_complex	1
TNFA_rna	1
TNFSF11	1
TNFSF11_TNFRSF11_complex	1
TPL2	1
TRADD	1
TRADD_TRAF2_RIP1_complex	1
TRAF2_RIP1_TRADD_TAK1_TAB1_TAB2_complex	1
TRAF2_TRAF6_complex	1
TRAF3	0
TRAF6	0
TRAF6_ECSIT_MEKK1_TAB1_TAB2_TAK1_complex	0
TRAF6_TAB1_TAB2_TAK1_complex	0
TRAF6_ubiquitinated	0
TRAM1	0
TRAM1_TRIF_complex	0
TRIF	1
TSG6	1
UEV1A_UBC13_complex	0,5
XIAP	1

Supplementary Table 3. The list of differentially expressed genes present in the RA M2 macrophage model that we identified using literature search and omics data analysis. The first column contains the DEGs HGNC names. The second and fifth columns contain their corresponding Boolean values observed in GSE97779 dataset and literature respectively.

DEG	Boolean value in GSE97779	Adjusted p_value	logFC	Boolean value based on literature	Reference
VEGFB	0	9,64E-06	-1,46		
PRLR	0	0,01306	-1,45		
MDM2	0	0,01706	-1,06		
CASP3	0	0,00542	-0,86		
TRAF6	0	0,00612	-0,73		
CREB1	0	0,03541	-0,66		
NRP2	0	0,03197	-0,65		
MAPK14	0	0,0243	-0,61		
SIRT1	0	0,0284	-0,61	0	25799392
SYK	0	0,015846	-0,53		
SMAD4	0	0,012	-0,46		
SMAD2	0	0,01636	-0,45		
BAD	0	0,02492	-0,42		
BCL3	1	0,04434	0,42		
HLA-B	1	0,02221	0,42		
CEBPB	1	0,00957	0,43		
SHC1	1	0,03891	0,44		
STAT6	1	0,02689	0,44		
BAX	1	0,02724	0,45	1	12634940
GNB1	1	0,00507	0,47		
IL6ST	1	0,01567	0,56		
CYLD	1	0,01375	0,61		
FCGR2A	1	0,02327	0,63	1	17521421
MAP2K2	1	0,00845	0,64		
RELA	1	0,01152	0,66		
CASP1	1	0,03031	0,71		
GNAI3	1	0,00098	0,72		
TGFB1	1	0,00044	0,78		
NFKBIE	1	0,01037	0,79		
CFLAR	1	0,0029	0,8	1	12228167
RXRA	1	0,0023	0,84		
IL4R	1	0,00047	0,86	1	7492352
PRKCD	1	0,0008	0,86		
NFKB1	1	0,00235	0,89	1	8630106
STAT3	1	0,00043	0,89		
HRAS	1	0,00388	0,94		
CASP7	1	0,00655	0,96		

LILRB1	1	0,0015	0,96		
NFAT5	1	0,01494	0,97		
NFKBIA	1	0,00021	0,98		
JAK1	1	0,00433	1		
PTPN6	1	2,86974E-05	1		
RAF1	1	0,01113	1		
SH2D1A	1	0,04778	1,01		
IL17RA	1	0,0044	1,02	1	19265168
MAP2K3	1	0,00258	1,03		
EFNB1	1	0,0094	1,09		
MAP2K1	1	0,0001	1,12		
PLCG2	1	0,00027	1,12		
XIAP	1	0,00215	1,12	1	19171073
HCK	1	5,23869E-05	1,13	1	17963503
IL12RB1	1	0,00343	1,29		
SOS1	1	0,01592	1,32		
HBEGF	1	0,04278	1,34	1	31068444
MCL1	1	0,00118	1,39	1	17009247
LIFR	1	0,01414	1,53		
PRKCQ	1	0,0037	1,53		
MYC	1	0,00027	1,66		
BCL2	1	0,01417	1,67		
BCL2L11	1	0,0833	1,78		
MAP2K6	1	0,02686	1,78		
PTK2	1	0,00073	1,87		
VEGFA	1	0,00429	1,9		
SMAD7	1	7,8882E-05	2,17		
JAK2	1	0,0011	2,3		
FAS	1	0,0005	2,45		
FCGR3A	1	2,49141E-05	2,77	1	22235253
FCGR1A	1	0,00011	3,32	1	17521421
DUSP1	1	5,59417E-07	4,17		
NLRP3	1	8,50103E-06	4,44		
KLF4	1	1,77E-08	4,66	1	29997611
BCL2L1				1	28118944
C5A				1	29220376
C5AR1				1	29220376
CCL21				1	21225692
CSF1				1	27036883
CSF1R				1	27036883
IL10				1	20001767

IL11				1	29327326
IL17F				1	19265168
IL17RC				1	19265168
IL23				1	25799392
IL34				1	22039170
IL4				1	7492352
IL8				1	10491366
MAPK3				1	17907188
TGFBR1				1	7614737
TGFBR2				1	7614737

Supplementary Table 4. List of nodes upstream the phenotypes of interest in the RA M2 macrophage model associated with their mean values over the fixpoints having the highest similarity score.

**Nodes**	**Mean values**
AKT1	0
AKT1_phosphorylated	0
apoptosis_M2_macrophage_phenotype	1
ASC	0,5
ASK1	1
BAD	0
BAX	1
BCL2_M2_macrophage_mitochondrion_membrane	1
BCL2_M2_macrophage_mitochondrion_membrane_active	0
Bcl2_rna	1
BCL2L1	0
BCL3_rna	1
Bim	1
C_EBPb_phosphorylated	1
c_Myc_rna	1
c5a	1
C5a_C5aR1_complex	1
Casp1	1
CASP3	1
CASP7	1
CASP8	1
CASP9	0
CCl21	1
CCL21_CCR7_complex	1
CD32a	1
CD40LG_ITGB1_ITGA1_complex	0,5
cFLIP	1
cMyc	1
cMyc_phosphorylated	1
col4a4	0,66666667

col4a5	0,66666667
CREB1_phosphorylated	0
CSF1_M2_macrophage__extracellular_space	1
CSF1_M2_macrophage__secreted_components	1
CSF1R	1
CSF1R_CSF1_complex	1
CSFR1R_IL34_complex	1
CXCR1_IL8_complex	1
CYLD	1
DAG_simple_molecule	1
DAXX	1
DUSP1	1
EFNB1_EPHB1_complex	1
ERK1_phosphorylated_M2_macrophage__cytoplasm	1
ERK1_phosphorylated_M2_macrophage_nucleus	1
FADD	1
FAS	1
FASL_FAS_complex	1
FASL_M2_macrophage__extracellular_space	1
FASL_M2_macrophage__secreted_components	1
FOXO1	1
GAB2_phosphorylated	1
GAS6	0,5
GAS6_MERTK_complex	0,5
GNAI3	1
GNB_GNG_complex	1
GNB_GNG_GNAI3_complex	1
Grb2	1
GSK3B	1
HBGEF	1
HCK	1
HLA_B_LILRB1_complex	1
HRAS	1
IKK_complex	1
IKK1_IKK2_complex	0,5
IKK1_phosphorylated	0,5
IKK2_phosphorylated	1
IL10_M2_macrophage__extracellular_space	1
IL10_M2_macrophage__secreted_components	1
IL10R1_IL10R2_complex	1
IL10R1_IL10R2_IL10_complex	1
IL11Ra_IL6ST_IL11_complex	1
IL17Ra_IL17Rc_IL17F_complex	1
IL23_M2_macrophage__extracellular_space	1
IL23_M2_macrophage__secreted_components	1
IL23R_IL12RB1_complex	1

IL23R_IL12RB1_IL23_complex	1
IL34	1
IL4	1
IL4_IL4Ra_complex	1
IL4R	1
IL8_M2_macrophage__extracellular_space	1
IL8_M2_macrophage__secreted_components	1
immune_complex_CD16a_complex	1
immune_complex_CD32a_complex	1
immune_complex_CD32b_complex	1
immune_complex_CD64_complex	1
immune_complex_complex	1
ITGB1_ITGA1_col4a_complex	1
ITGB1_ITGA1_complex	1
JAK1	1
JAK1_TYK2_complex	1
JAK2	1
klf4	1
LIFR_IL6ST_CTF1_complex	1
mcl1	0
Mcl1_rna	0
MDM2_phosphorylated	0
MEK1_phosphorylated	1
MEK2_phosphorylated	1
MKK3_phosphorylated	1
MKK6_phosphorylated	1
MSK1_phosphorylated	0
NFAT5_phosphorylated	1
NFKB1_TPL2_complex	1
NFKBIA_RELA_NFKB1_complex	1
NLRP3	1
NLRP3_INFLAMMASOME_complex	0,5
p15_rna	1
p38_MAP_KINASE_phosphorylated_M2_macrophage__cytoplasm	0
p38_MAP_KINASE_phosphorylated_M2_macrophage_nucleus	0
p53_phosphorylated_M2_macrophage__cytoplasm	1
p53_phosphorylated_M2_macrophage_nucleus	1
PI3K	0
PIK3AP1_phosphorylated	0
PIP2_simple_molecule	1
PLCG2	1
PRKCD	1
Prkcd	1
PRKCQ	1
PRL	0

PRL_PRLR_complex	0
proliferation_survival_M2_macrophage_phenotype	0
PTK2	1
PTPN6	1
RAF1	1
RBL1_E2F4_DP1_complex	0
RELA_NFKB1_complex_M2_macrophage__cytoplasm	1
RELA_NFKB1_complex_M2_macrophage_nucleus	1
RELA_NFKB1_NFKBIE_complex	1
RXRa_NUR77_complex	1
SH2D1A	1
Shc_phosphorylated	1
SHIP1	1
Sirt1	0
SMAD2_phosphorylated	0
SMAD2_SARA_complex	0
SMAD2_SMAD4_complex	0
SMAD4	0
SMAD7	1
SOS1	1
Src	1
STAT3	1
STAT3_STAT3_complex	1
STAT6	1
STAT6_STAT6_complex	1
SYK	0
Syk_phosphorylated	1
TAK1_phosphorylated	1
TGFB1_M2_macrophage__extracellular_space	1
TGFB1_M2_macrophage__secreted_components	1
TGFBR1_TGFBR2_complex	1
TGFBR1_TGFBR2_TGFB1_complex	1
TPL2	1
TRAF3IP2_phosphorylated	1
TRAF6_ubiquitinated	0
VEGFa_M2_macrophage__extracellular_space	1
VEGFa_M2_macrophage__secreted_components	1
Vegfa_rna	1
Vegfb_M2_macrophage__extracellular_space	0
Vegfb_M2_macrophage__secreted_components	0
Vegfc_M2_macrophage__extracellular_space	0
Vegfc_M2_macrophage__secreted_components	0
vegfc_vegfr3_complex	0
VegfR1	0,5
VegfR1_Vegfa_complex	0,5
VegfR1_vegfb_complex	0

VegfR2_vegfc_nrp2_complex	0
XIAP	1

Supplementary Table 5. Therapeutic drug targets in the RA M1 macrophage model

**Therapeutic target**
SRC
SIRT1
MAPK14
AKT1
PTPN6
TLR9
TLR8
TLR7
MCL1
MDM2
JAK2
JAK1
CSF2
IL23R
SYK
RAF1
CCR2

BCL2L1
CASP3
CASP8
FAS
ACVR2B
IRAK4
BCL2
C5AR1
TBK1
GALR2
IRAK1
TYK2
CASP1
CASP7
NLRP3
PRKCD
XIAP
PTK2
PPIA
TLR2

TLR4
PRKCQ
IL6R
IL6ST
TNF
DUSP1
JUN
NFKB1
RELA
CXCR1
MAPK3
ITGB1
MYC
STAT3
RAC1
IL1B
CCL2
RHOA
STAT1
IFNG

CD40LG
TNFSF11
IL6
INHBA
INHBB
NOTCH1
CSF2RB
IL12A
ITGA1
NFKBIA
IFNAR2
SMAD7
ACVR2A
IL18

Supplementary Table 6. Therapeutic drug targets in the RA M2 macrophage model

**Therapeutic Target**
SRC
GSK3B
SIRT1
MAPK14

AKT1
PTPN6
HCK
MCL1
MDM2
JAK2
CSF1R
JAK1
VEGFA
IL23R
IL4R
SYK
RAF1
TGFBR1
BCL2L1
CASP3
CASP8
FAS
BCL2
C5AR1

TYK2
RXRA
MERTK
TGFBR2
CASP1
CASP7
NLRP3
PRKCD
XIAP
PTK2
PRKCQ
IL6ST
CASP9
DUSP1
NFKB1
RELA
CXCR1
MAPK3
ITGB1
MYC

STAT3
CD40LG
KLF4
PRLR
CSF1
TGFB1
LIFR
IL4
ITGA1
GAS6
VEGFC
NFKBIA
BAX
SMAD7
IL17RA
IL17RC
